# Optically Switchable NIR Photoluminescence of PbS Semiconducting Nanocrystals using Diarylethene Photoswitches

**DOI:** 10.1021/jacs.2c07102

**Published:** 2022-09-23

**Authors:** Lili Hou, Rasmus Ringström, Andrew B. Maurer, Maria Abrahamsson, Joakim Andréasson, Bo Albinsson

**Affiliations:** Department of Chemistry and Chemical Engineering, Chalmers University of Technology, Gothenburg 41296, Sweden

## Abstract

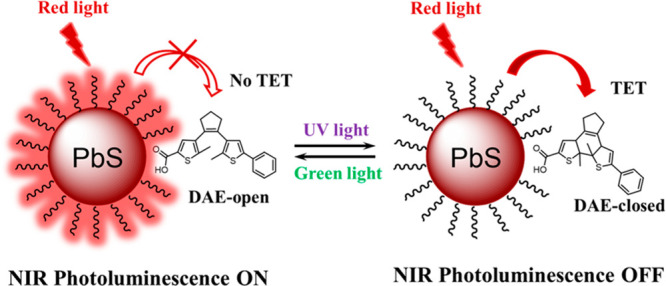

Precisely modulated photoluminescence (PL) with external control is highly demanded in material and biological sciences. However, it is challenging to switch the PL *on* and *off* in the NIR region with a high modulation contrast. Here, we demonstrate that reversible *on* and *off* switching of the PL in the NIR region can be achieved in a bicomponent system comprised of PbS semiconducting nanocrystals (NCs) and diarylethene (DAE) photoswitches. Photoisomerization of DAE to the ring-closed form upon UV light irradiation causes substantial quenching of the NIR PL of PbS NCs due to efficient triplet energy transfer. The NIR PL fully recovers to an *on* state upon reversing the photoisomerization of DAE to the ring-open form with green light irradiation. Importantly, fully reversible switching occurs without obvious fatigue, and the high PL *on*/*off* ratio (>100) outperforms all previously reported assemblies of NCs and photoswitches.

Modulating photoluminescence (PL) between a “dark” *off* state and a “bright” *on* state is of great interest for the application of smart response materials,^1^ optical memory,^2,3^ super-resolution imaging,^4^ and medical probing.^5^ Among many external stimuli used to switch PL, noninvasive photonic control is a waste-free method that offers the advantages of high spatial and temporal resolution and the the convenience of remotely tuning the irradiation wavelength and intensity. A simple way to implement an optically switchable PL system (OSPLS) is to combine a highly emissive material with a photochromic molecular switch,^6−8^ generally referred to as photoswitch. Colloidal semiconducting nanocrystals (NCs),^9,10^ often referred to as quantum dots, with outstanding performances such as bright PL and size-tunable emission wavelengths are ideal PL materials for constructing OSPLS. Photoswitches, whose two isomers can interconvert upon irradiation at different wavelengths, can be used in combination with NCs.^11−13^ The photoswitches are typically bound to the surface of NCs to construct OSPLS in which only one isomer can quench the PL of the NCs, hence the conversion between the isomers upon light irradiation results in the modulation of the PL intensity.

The mechanisms of reported OSPLSs that use NCs and photoswitches have been based on Förster resonance energy transfer (FRET)^14−20^ and photoinduced electron transfer (PET).^21−23^ Thus far, OSPLSs have displayed modulated emission only in the visible region and comparably low PL *on*/*off* ratios. Here, we present a highly efficient OSPLS comprised of PbS NCs and diarylethene (DAE) photoswitches, where the NIR PL of PbS NCs can be reversibly switched *on* and *off* when the DAE-open and DAE-closed isomers interconvert upon light irradiation. The intensity of the NIR PL in the “dark” *off* state is low enough to yield an *on*/*off* ratio larger than 100, while the emission of the PbS NCs is essentially unaffected in the *on* state. The key to achieving the optically switchable NIR PL of our design is the large difference between the excited triplet-state energies of DAE-open and DAE-closed. The triplet energy transfer (TET) that induces NIR PL quenching exclusively occurs from PbS NCs to DAE-closed, as the triplet excited state of DAE-open is much too high to allow this process.

The PbS NCs and DAE were synthesized according to previous reports.^24,25^Figure 1A shows the absorption and PL spectra of PbS NCs in toluene. The first absorption band of PbS NCs has a peak maximum around 750 nm, and the emission is centered in the NIR region with a peak maximum at 820 nm. The average size of the PbS NCs is approximately 2–3 nm as determined by scanning transmission electron microscopy (STEM) (see the inset of Figure 1A). Figure 1B presents absorption spectra and the chemical structures of DAE-open and DAE-closed, which can be reversibly interconverted into each other under UV and visible-light irradiation. The carboxylic acid functional group allows the DAEs to anchor to the surface of PbS NCs. UV light irradiation of DAE-open results in the appearance of two new absorption bands with maxima at 360 and 545 nm, clearly showing the formation of DAE-closed. The photostationary state (PSS) of DAE in solution under UV light irradiation consists of 90% DAE-closed and 10% DAE-open as determined by HPLC (see the SI). Upon the subsequent irradiation of the solution at PSS with green light, the spectrum fully recovers to the initial state. DAE shows some photochromic fatigue over the switching cycles upon UV and green irradiation (see Figure S1). It should be noted that neither DAE-open nor DAE-closed emits light in the NIR region.

**Figure 1:**
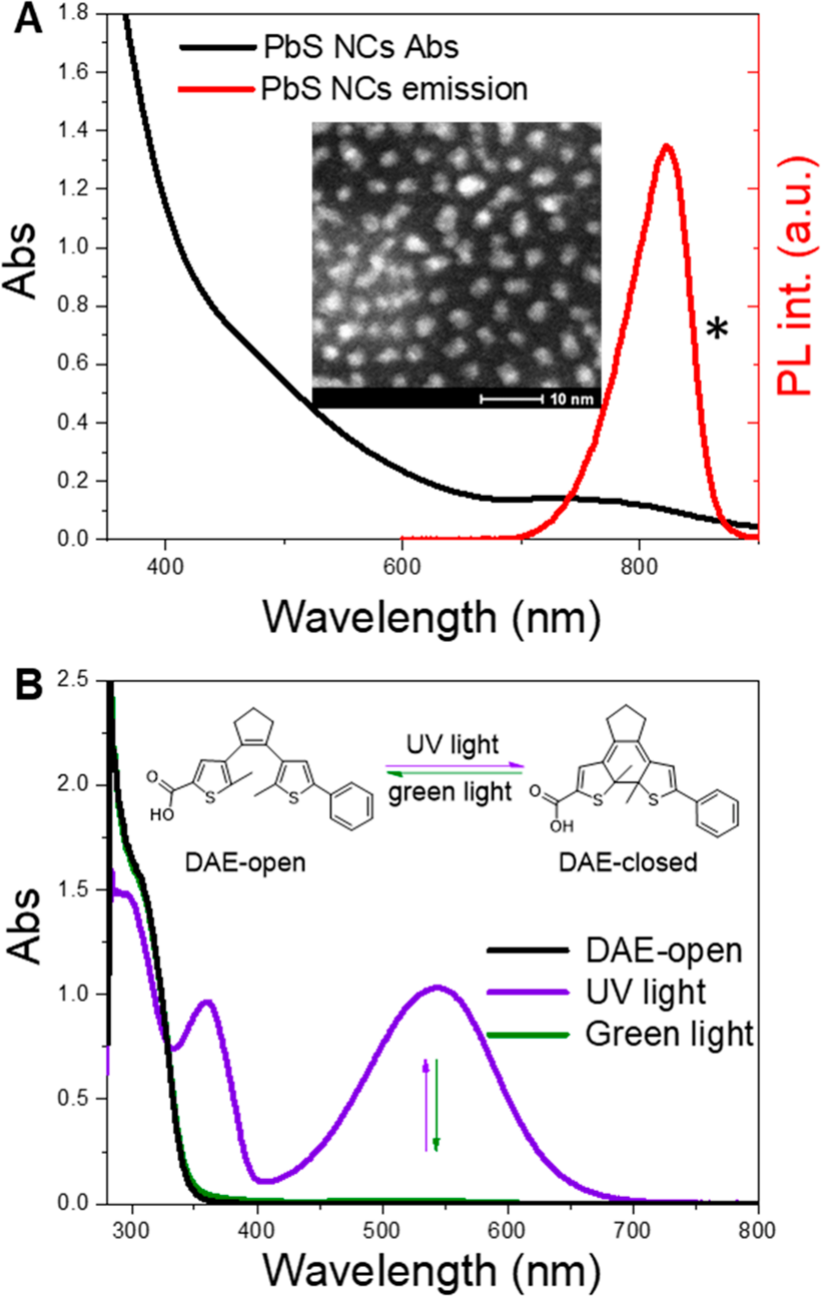
Absorption spectra of PbS NCs and DAE. (A) Absorption and PL spectra of PbS NCs (1 μM) in toluene. *The distortion from the Gaussian band shape is caused by a drop in instrument sensitivity for λ > 850 nm. (Inset) STEM image of PbS NCs. (B) Absorption spectra of 100 μM DAE-open in toluene before irradiation, after UV (302 nm, 60 s) light irradiation, and after subsequent green light irradiation (523 nm, 60 s).

The photochromic properties of DAE were retained after it was mixed with PbS NCs. Figure 2A shows the absorption spectra of a solution of 1.5 μM PbS NCs mixed with 100 μM DAE in deaerated toluene. UV light irradiation induces the formation of the typical absorption band in the visible region for DAE-closed, which disappears with green light irradiation. Washing and precipitating the mixed solution confirms that DAEs are bound to the surface of PbS NCs and allows an estimation of the number of DAEs bound per NC, which is detailed in section 3 of the SI. After mixing with DAE, the NIR PL of PbS NCs can be switched *on* and *off* upon alternating exposure to UV and visible light, as shown in Figure 2B. It should be noted that the light at 680 nm used for the PbS NC emission readout is not absorbed by any isomeric form of DAE, implying that this process is orthogonal to DAE photoisomerization. The PL intensity of the DAE-open mixed solution is virtually the same as that of PbS NCs alone at the same conditions, indicating no significant interaction between DAE-open and the excited state of PbS NCs. In contrast, the formation of DAE-closed upon UV irradiation yields in a 99% quenching of the PL intensity. Subsequent irradiation with green light converts DAE back to the open form, resulting in the full recovery of the PL spectra to the initial state. The PL *on*/*off* ratio is >100 for 1.5 μM PbS NCs with 67 equiv of DAE. Such a large degree of modulation is much higher than those for previously reported systems comprised of NCs and photoswitches. For instance, CdSe/ZnS NCs surface-bound with 90–100 equiv of spiropyran photoswitches showed a PL *on*/*off* ratio of ca. 10 via FRET control,^14^ and the assembly of CdSe NCs with 600 equiv of furyfulgide photoswitches had a maximum PL *on*/*off* ratio of 25 via PET control.^23^ The PL lifetimes of PbS NCs were also examined using time-resolved PL measurements (see Figure S4). PbS NCs show a PL lifetime of about 2.8 μs, which decreases slightly to 2.7 μs after the NCs are mixed with DAE-open. Upon isomerization to DAE-closed, the PL lifetime of PbS NCs dramatically decreases below the resolution of our instrument response function (0.7 μs) in the NIR region, which is consistent with the substantial quenching observed in the steady-state measurements.

**Figure 2:**
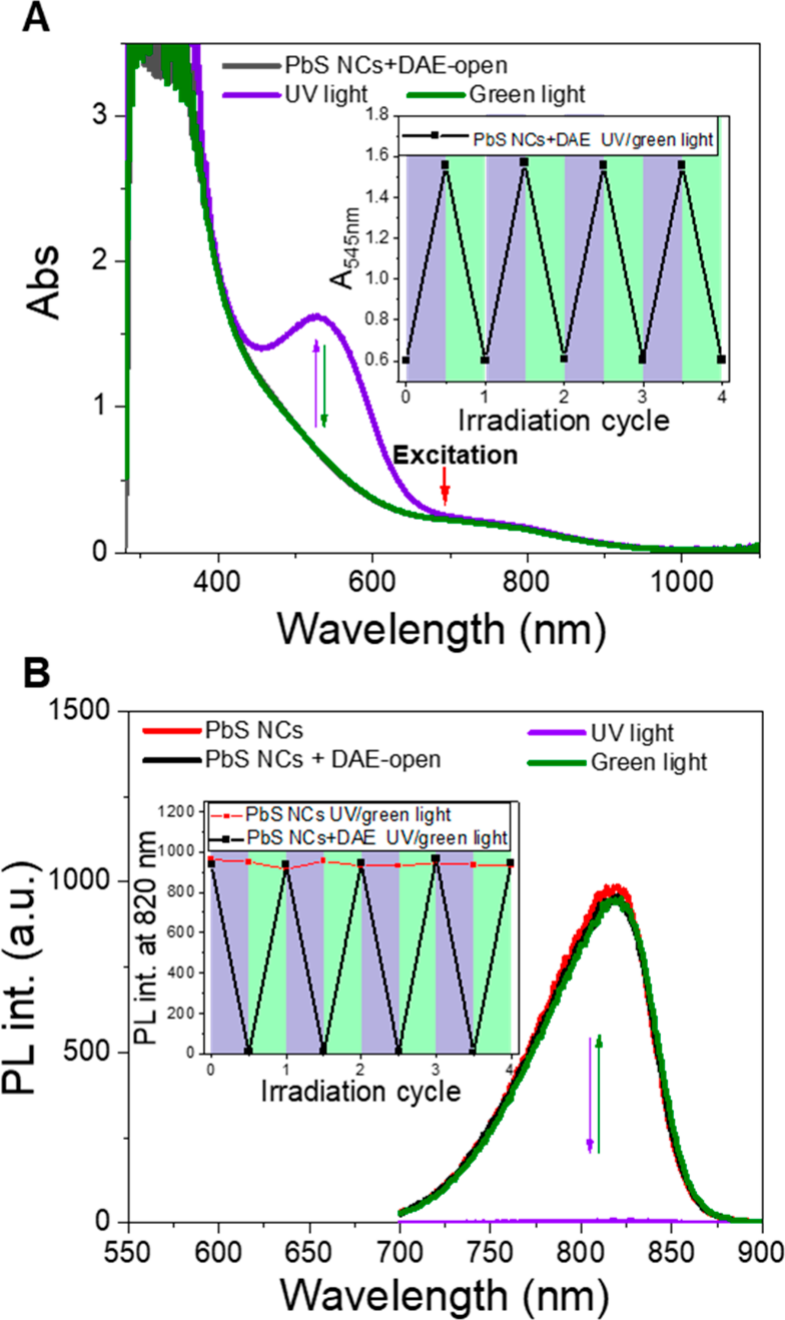
Light modulation of the absorption and PL spectra of PbS NCs mixed with DAEs. (A) Absorption spectra of the mixed solution before and after 60 s of UV light irradiation and after 400 s of green light irradiation. (B) PL spectra of PbS NCs alone and PbS NCs mixed with DAEs before and after light irradiation. (Inset) Absorbance and PL intensity over four irradiation cycles with UV and green light.

The photoswitching of the NIR PL of our design is reversible and fatigue-resistant. Changes in the absorbance and the PL intensity were monitored over four cycles with alternating UV and green light irradiation (see insets of Figure 2). Although DAE itself shows some photoswitching fatigue because of photodegradation under UV light irradiation, the switching behavior is stable over several cycles when it is loaded together with PbS NCs. This can be attributed to the high molar extinction coefficient of PbS NCs, which attenuates the UV light; only ∼14% of the UV photons are absorbed by DAE-open (detailed in section 4 of the SI). Under the same illumination conditions (302 nm, 60 s), 50% of the DAE molecules convert to the closed form in the mixture compared to 90% conversion for a solution containing DAE alone. It should be noted that 50% conversion of DAE is enough to efficiently quench the NIR PL of PbS NCs. As a control measurement, a sample prepared with PbS NCs alone did not show any light-induced modulation of the PL intensity (red curve in the inset of Figure 2B).

Femtosecond transient absorption (fsTA) spectroscopy was performed to gain further insight into the mechanism of the PL quenching (see Figure 3). The model fitting and experimental decay data for a few selected wavelengths are shown in Figure S5. For PbS NCs alone, the broad absorption band observed in the visible region is consistent with previously reported PbS NCs.^26−28^ Our global analysis with a sequential two-component model resulted in two similar spectral components (see the top right panel in Figure 3B). The first component decayed symmetrically over the entire spectral range with a time constant of ∼3.8 ps, which was previously ascribed to multiple exciton annihilation^28,29^ or hot carrier cooling.^30^ The second component has a lifetime that is much longer than our delay stage (10 ns) and is likely the excited state of PbS NCs, which decays on the microsecond time scale (vide supra).^28^ fsTA spectra and the corresponding spectral components of PbS NCs mixed with DAE-open are essentially identical to those of PbS NCs alone. This indicates that neither energy transfer nor electron transfer occurs from PbS NCs to DAE-open, which corresponds well with the unquenched emission of PbS NCs. However, when PbS NCs are mixed with DAE-closed, the spectral evolution changes drastically. The short-lived component shows a lifetime similar to that of PbS NCs alone, while the lifetime of the broad and long-lived PbS NCs excited state absorption decreases to 2.7 ns, showing that the excited state of PbS NCs is strongly quenched. As the excited state of PbS NCs decays, a new species with two distinct absorption bands centered at 488 and 560 nm forms. The new species corresponds to the acceptor state of DAE-closed, which we assign to the triplet excited state (section 9 of the SI).

**Figure 3:**
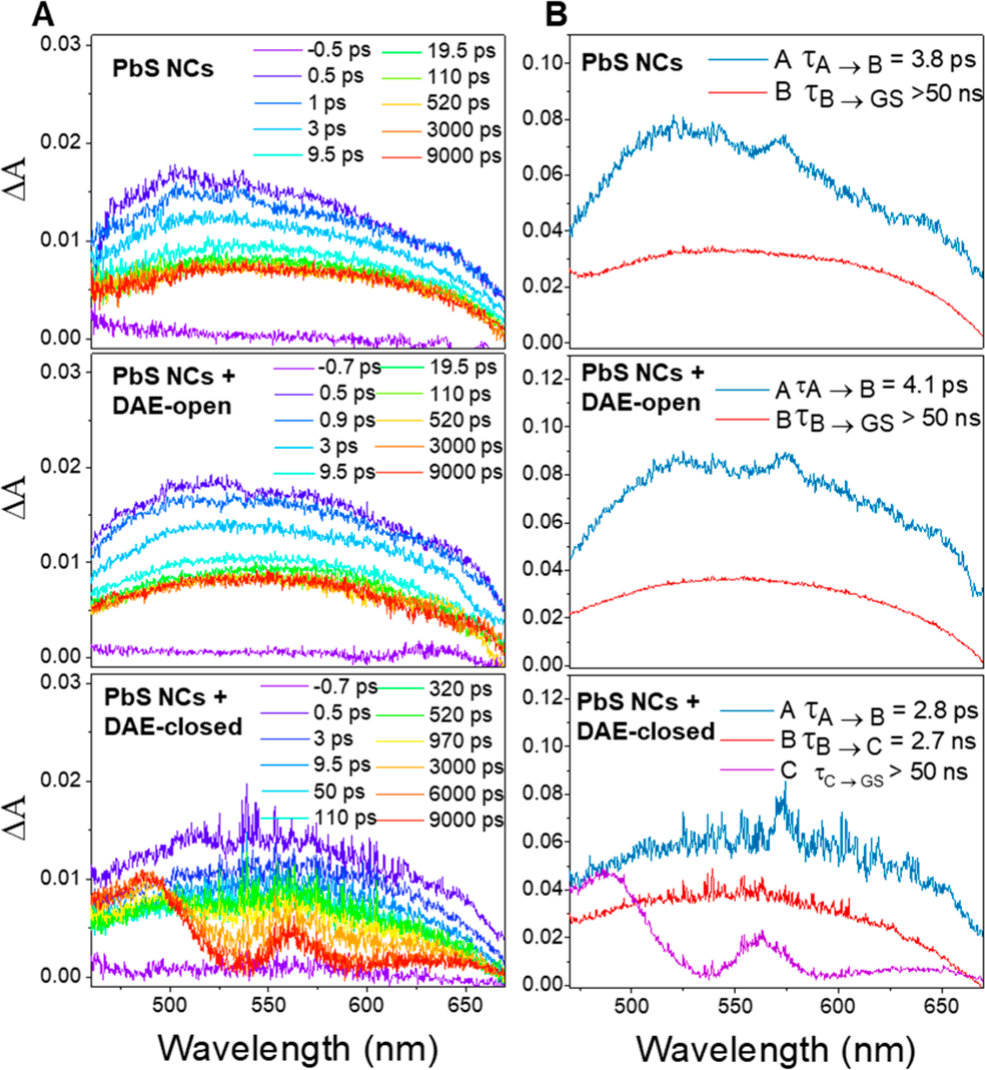
(A) Transient absorption spectra and (B) corresponding evolution-associated spectra of PbS NCs alone and PbS NCs mixed with DAEs.

The suggested quenching mechanism and triplet energy levels are summarized in Figure 4. According to density functional theory (DFT, see SI) calculation, the lowest triplet excited state (T_1_) energies of DAE-open and DAE-closed are 2.65 and 0.89 eV, respectively. NCs exhibit a strong spin–orbit coupling with substantial mixing of the singlet and triplet states,^31,32^ and both the bright and dark exciton states can contribute to TET.^33,34^ The excited state of the synthesized PbS NCs can be estimated to be 1.65 eV from the peak of the first excitonic transition. As such, the T_1_ state of DAE-open is much higher in energy than the PbS NCs. Conversely, the T_1_ state of DAE-closed is 740 meV lower than that of PbS NCs; thus, TET can only occur from PbS NCs to DAE-closed and not DAE-open. The large TET driving force between PbS NCs and DAE-closed is the key to achieving the high PL *on/off* ratio of our system.

**Figure 4:**
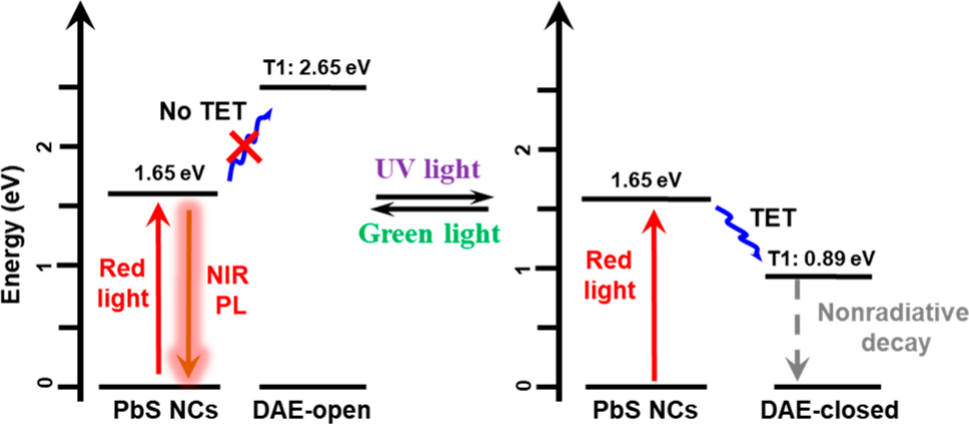
Schematic illustration of the light modulation of the NIR PL based on TET between PbS NCs and DAE.

FRET quenching can be excluded in our design, as there is no overlap between the PL spectrum of PbS NCs and the absorption spectra of either form of DAE. The final quenching mechanism to consider is PET, which is discussed in detail in section 8 of the SI. Briefly, there is only a small driving force for PET from PbS NCs to DAE-closed, which is unlikely to cause such highly efficient PL quenching. Additionally, component C in Figure 3 cannot be modeled by the absorption spectra of either the radical anion or the radical cation obtained through spectroelectrochemistry (Figure S9). We therefore conclude that TET rather than PET is the main mechanism responsible for the efficient PL quenching observed herein.

In summary, we have designed an OSPLS comprised of PbS NCs and DAE photoswitches in which the NIR PL can be efficiently and reversibly switched *on* and *off* by illuminating the mixture with distinct wavelengths. The high PL *on*/*off* ratio (>100) significantly outperforms all previously reported assemblies of NCs and photoswitches. The switching capability in the NIR region is primarily attributed to the mechanism of triplet energy transfer, which is only energetically possible from PbS NCs to the triplet excited state of DAE-closed, not DAE-open. The large difference between the excited triplet state energies of the DAE derivatives in the open form and the closed form can in principle be fine-tuned over a wide spectral window. By choosing appropriate NCs in combination with visible or NIR triggered DAE photoswitches,^35−37^ it should be possible to observe efficient NIR PL switching that is activated without using UV light. Our approach will enable further optimization in optically switchable electronic devices and optical memory and could offer precise control in super-resolution imaging toward biological applications.

## Supporting Information Available

Detailed description of the synthesis of PbS NCs, descriptions of experimental setups, computational details, additional spectroscopic data, and discussions regarding PET and TET (PDF)

## References

[ref1] Mutai T.; Satou H.; Araki K. Reproducible on-off switching of solid-state luminescence by controlling molecular packing through heat-mode interconversion. Nat. Mater. 2005, 4 (9), 685–687. 10.1038/nmat1454. 16113683

[ref2] Irie M.; Fukaminato T.; Sasaki T.; Tamai N.; Kawai T. Organic chemistry: A digital fluorescent molecular photoswitch. Nature 2002, 420 (6917), 759–760. 10.1038/420759a. 12490936

[ref3] Sun H. B.; Liu S. J.; Lin W. P.; Zhang K. Y.; Lv W.; Huang X.; Huo F. W.; Yang H. R.; Jenkins G.; Zhao Q.; Huang W. Smart responsive phosphorescent materials for data recording and security protection. Nat. Commun. 2014, 5, 3601 10.1038/ncomms4601. 24710282

[ref4] Li C.; Yan H.; Zhao L. X.; Zhang G. F.; Hu Z.; Huang Z. L.; Zhu M. Q. A trident dithienylethene-perylenemonoimide dyad with super fluorescence switching speed and ratio. Nat. Commun. 2014, 5, 5709 10.1038/ncomms6709. 25502396

[ref5] Kobayashi H.; Ogawa M.; Alford R.; Choyke P. L.; Urano Y. New Strategies for Fluorescent Probe Design in Medical Diagnostic Imaging. Chem. Rev. 2010, 110 (5), 2620–2640. 10.1021/cr900263j. 20000749 PMC3241938

[ref6] Heilemann M.; Dedecker P.; Hofkens J.; Sauer M. Photoswitches: Key molecules for subdiffraction-resolution fluorescence imaging and molecular quantification. Laser Photonics Rev. 2009, 3 (1–2), 180–202. 10.1002/lpor.200810043.

[ref7] Szymanski W.; Beierle J. M.; Kistemaker H. A. V.; Velema W. A.; Feringa B. L. Reversible Photocontrol of Biological Systems by the Incorporation of Molecular Photoswitches. Chem. Rev. 2013, 113 (8), 6114–6178. 10.1021/cr300179f. 23614556

[ref8] Zhang X. Y.; Hou L. L.; Samori P. Coupling carbon nanomaterials with photochromic molecules for the generation of optically responsive materials. Nat. Commun. 2016, 7, 11118 10.1038/ncomms11118. 27067387 PMC4832057

[ref9] Alivisatos A. P. Semiconductor clusters, nanocrystals, and quantum dots. Science 1996, 271 (5251), 933–937. 10.1126/science.271.5251.933.

[ref10] Burda C.; Chen X. B.; Narayanan R.; El-Sayed M. A. Chemistry and properties of nanocrystals of different shapes. Chem. Rev. 2005, 105 (4), 1025–1102. 10.1021/cr030063a. 15826010

[ref11] Molecular Switches, 1st ed.; Feringa B. L., Ed.; Wiley-VCH: Weinheim, Germany, 2001.

[ref12] Russew M. M.; Hecht S. Photoswitches: From Molecules to Materials. Adv. Mater. 2010, 22 (31), 3348–3360. 10.1002/adma.200904102. 20422653

[ref13] Irie M. Diarylethenes for memories and switches. Chem. Rev. 2000, 100 (5), 1685–1716. 10.1021/cr980069d. 11777416

[ref14] Zhu L. Y.; Zhu M. Q.; Hurst J. K.; Li A. D. Q. Light-controlled molecular switches modulate nanocrystal fluorescence. J. Am. Chem. Soc. 2005, 127 (25), 8968–8970. 10.1021/ja0423421. 15969571 PMC1435833

[ref15] Diaz S. A.; Giordano L.; Jovin T. M.; Jares-Erijman E. A. Modulation of a Photoswitchable Dual-Color Quantum Dot containing a Photochromic FRET Acceptor and an Internal Standard. Nano Lett. 2012, 12 (7), 3537–3544. 10.1021/nl301093s. 22663176

[ref16] Diaz S. A.; Gillanders F.; Jares-Erijman E. A.; Jovin T. M. Photoswitchable semiconductor nanocrystals with self-regulating photochromic Forster resonance energy transfer acceptors. Nat. Commun. 2015, 6, 6036 10.1038/ncomms7036. 25592060

[ref17] Schmidt L. C.; Edelsztein V. C.; Spagnuolo C. C.; Di Chenna P. H.; Galian R. E. Light-responsive hybrid material based on luminescent core-shell quantum dots and steroidal organogel. J. Mater. Chem. C 2016, 4 (29), 7035–7042. 10.1039/C6TC02265K.

[ref18] Yano N.; Yamauchi M.; Kitagawa D.; Kobatake S.; Masuo S. Photoluminescence On/Off Switching of a Single Colloidal Quantum Dot Using Photochromic Diarylethene. J. Phys. Chem. C 2020, 124 (31), 17423–17429. 10.1021/acs.jpcc.0c05030.

[ref19] Zhu L. Y.; Wu W. W.; Zhu M. Q.; Han J. J.; Hurst J. K.; Li A. D. Q. Reversibly photoswitchable dual-color fluorescent nanoparticles as new tools for live-cell imaging. J. Am. Chem. Soc. 2007, 129 (12), 3524–3526. 10.1021/ja068452k. 17335209 PMC2546355

[ref20] Su J.; Fukaminato T.; Placial J. P.; Onodera T.; Suzuki R.; Oikawa H.; Brosseau A.; Brisset F.; Pansu R.; Nakatani K.; Metivier R. Giant Amplification of Photoswitching by a Few Photons in Fluorescent Photochromic Organic Nanoparticles. Angew. Chem., Int. Ed. 2016, 55 (11), 3662–3666. 10.1002/anie.201510600. 26821998

[ref21] Saeed S.; Yin J.; Khalid M. A.; Channar P. A.; Shabir G.; Saeed A.; Nadeem M. A.; Soci C.; Iqbal A. Photoresponsive azobenzene ligand as an efficient electron acceptor for luminous CdTe quantum dots. J. Photochem. Photobiol. A 2019, 375, 48–53. 10.1016/j.jphotochem.2019.02.007.

[ref22] Bang J.; Park J.; Velu R.; Yoon E.; Lee K.; Cho S.; Cha S.; Chae G.; Joo T.; Kim S. Photoswitchable quantum dots by controlling the photoinduced electron transfers. Chem. Commun. 2012, 48 (73), 9174–9176. 10.1039/c2cc34002j. 22872047

[ref23] Padgaonkar S.; Eckdahl C. T.; Sowa J. K.; Lopez-Arteaga R.; Westmoreland D. E.; Woods E. F.; Irgen-Gioro S.; Nagasing B.; Seideman T.; Hersam M. C.; Kalow J. A.; Weiss E. A. Light-Triggered Switching of Quantum Dot Photoluminescence through Excited-State Electron Transfer to Surface-Bound Photochromic Molecules. Nano Lett. 2021, 21 (1), 854–860. 10.1021/acs.nanolett.0c04611. 33395307

[ref24] Hines M. A.; Scholes G. D. Colloidal PbS nanocrystals with size-tunable NIR emission: Observation of post-synthesis self-narrowing of the particle size distribution. Adv. Mater. 2003, 15 (21), 1844–1849. 10.1002/adma.200305395.

[ref25] Branda N. R.; Myles A. J., Novel Photochromic Polymers and Methods of Synthesizing Same. WO 2002006361 A2, 2002.

[ref26] Yang Y.; Rodríguez-Córdoba W.; Lian T. Ultrafast Charge Separation and Recombination Dynamics in Lead Sulfide Quantum Dot–Methylene Blue Complexes Probed by Electron and Hole Intraband Transitions. J. Am. Chem. Soc. 2011, 133 (24), 9246–9249. 10.1021/ja2033348. 21615168

[ref27] Yang Y.; Rodríguez-Córdoba W.; Xiang X.; Lian T. Strong Electronic Coupling and Ultrafast Electron Transfer between PbS Quantum Dots and TiO2 Nanocrystalline Films. Nano Lett. 2012, 12 (1), 303–309. 10.1021/nl2035783. 22182013

[ref28] Garakyaraghi S.; Mongin C.; Granger D. B.; Anthony J. E.; Castellano F. N. Delayed Molecular Triplet Generation from Energized Lead Sulfide Quantum Dots. J. Phys. Chem. Lett. 2017, 8 (7), 1458–1463. 10.1021/acs.jpclett.7b00546. 28300410

[ref29] Yang Y.; Rodríguez-Córdoba W.; Lian T. Multiple Exciton Generation and Dissociation in PbS Quantum Dot-Electron Acceptor Complexes. Nano Lett. 2012, 12 (8), 4235–4241. 10.1021/nl301847r. 22757981

[ref30] Ellingson R. J.; Beard M. C.; Johnson J. C.; Yu P. R.; Micic O. I.; Nozik A. J.; Shabaev A.; Efros A. L. Highly efficient multiple exciton generation in colloidal PbSe and PbS quantum dots. Nano Lett. 2005, 5 (5), 865–871. 10.1021/nl0502672. 15884885

[ref31] Jiang Y. S.; Wang C.; Rogers C. R.; Kodaimati M. S.; Weiss E. A. Regio- and diastereoselective intermolecular [2 + 2] cycloadditions photocatalysed by quantum dots. Nat. Chem. 2019, 11 (11), 1034–1040. 10.1038/s41557-019-0344-4. 31654049 PMC6820707

[ref32] Efros A. L.; Rosen M.; Kuno M.; Nirmal M.; Norris D. J.; Bawendi M. Band-edge exciton in quantum dots of semiconductors with a degenerate valence band: Dark and bright exciton states. Phys. Rev. B 1996, 54 (7), 4843–4856. 10.1103/PhysRevB.54.4843. 9986445

[ref33] Mongin C.; Garakyaraghi S.; Razgoniaeva N.; Zamkov M.; Castellano F. N. Direct observation of triplet energy transfer from semiconductor nanocrystals. Science 2016, 351 (6271), 369–372. 10.1126/science.aad6378. 26798011

[ref34] Jin T.; He S.; Zhu Y. F.; Egap E.; Lian T. Q. Bright State Sensitized Triplet Energy Transfer from Quantum Dot to Molecular Acceptor Revealed by Temperature Dependent Energy Transfer Dynamics. Nano Lett. 2022, 22 (10), 3897–3903. 10.1021/acs.nanolett.2c00017. 35561343

[ref35] Bleger D.; Hecht S. Visible-Light-Activated Molecular Switches. Angew. Chem., Int. Ed. 2015, 54 (39), 11338–11349. 10.1002/anie.201500628. 26096635

[ref36] Li Z. Y.; He C. J.; Lu Z. Q.; Li P. S.; Zhu Y. P. Recent progress in all-visible-light-triggered diarylethenes. Dyes Pigm 2020, 182, 108623 10.1016/j.dyepig.2020.108623.

[ref37] Zhang Z.; Wang W.; O’Hagan M.; Dai J.; Zhang J.; Tian H. Stepping Out of the Blue: From Visible to Near-IR Triggered Photoswitches. Angew. Chem., Int. Ed. 2022, 61 (31), e202205758 10.1002/anie.202205758. 35524420

