# Supplementary material for: Optically Switchable NIR Photoluminescence of PbS Semiconducting Nanocrystals using Diarylethene Photoswitches
Source: J Am Chem Soc. 2022 Sep 23;144(39):17758–62. doi: 10.1021/jacs.2c07102 (PMC9545151; doi:10.1021/jacs.2c07102)
Supplement: Supplementary file 1 — ja2c07102_si_001.pdf [file ja2c07102_si_001.pdf]

<u>**Supplementary Information**</u>

Optically Switchable NIR Photoluminescence of PbS Semiconducting Nanocrystals using Diarylethene Photoswitches

Lili Hou, † ⊥ § * Rasmus Ringström,† Andrew B. Maurer, † Maria Abrahamsson, † Joakim Andréasson,† Bo Albinsson†*

AUTHOR ADDRESS

† Department of Chemistry and Chemical Engineering, Chalmers University of Technology, Gothenburg, 41296, Sweden

⊥ Present address: School of Precision Instruments and Optoelectronics Engineering, Tianjin University, Tianjin 300072, China.

§ Present address: Key Laboratory of Optoelectronics Information Technology, Ministry of Education, Tianjin 300072, China.

*Corresponding author e-mails: lilihou@tju.edu.cn; balb@chalmers.se

Contents

1. **Methods** S1
2. **Photochromic DAE** S3
3. **Steady-state characterization of DAE bound to PbS NCs** S4
4. **Determination of the proportion of UV photon absorbed by DAEs in the mixture** S4
5. **Photoluminescence lifetime measurement** S5
6. **Transient absorption decay traces** S6
7. **Cyclic voltammetry of DAEs** S6
8. **Discussion on photoinduced electron transfer** S7
9. **Discussion on triplet spectroscopic signature of DAE-closed** S8
10. **References** S10

1. Methods

Chemicals

Analytical reagent grade toluene was purchased from VWR and used without further purification for spectroscopic measurements. Lead oxide (99.999%), 1-octadecene (ODE, 90%), and hexamethyldisilathiane ($(TMS)_2S$) were purchased from Aldrich. Oleic acid (90%) was purchased from Alfa Aesar.

PbS NCs synthesis

PbS NCs were synthesized according to previous reports.[1-2] In short, 360 mg of PbO (1.6 mmol), 1 ml of oleic acid (3.2 mmol) and 15 ml of ODE were mixed in a 50 ml three-necked flask, which was degassed under vacuum

at 120 °C for 1 hour. After that, the solution was cooled down to room temperature under N_2. The sulfur precursor was prepared by dissolving168 µl of $(TMS)_2S$ (0.8 mmol) in 8 ml of ODE, and degassing under N_2 for 1 hour at room temperature. The sulfur precursor was then injected into the lead oleate solution at 110 °C and reacted for 1 hour. The reaction was monitored by UV-Vis absorption. The obtained PbS NCs were further purified via precipitation in argon-sparged acetonitrile/acetone. This purification was repeated two additional times.

Sample preparation

All photophysical measurements were carried out in toluene or acetonitrile using a 10 or 2 mm path quartz cuvette. The samples containing PbS NCs were prepared by sparging argon for at least 15 minutes.

Spectroscopy

UV-visible absorption spectra were measured using a Cary 50 UV-vis-NIR spectrophotometer. Steady-state photoluminescence spectra were recorded on a Cary Eclipse fluorescence spectrophotometer. Time-resolved photoluminescence lifetime measurements were carried out on a home-built system, in which an Nd:YAG laser (10 ns FWHM pulsewidth, 10 Hz, Spectra-Physics, Quanta-Ray) equipped with an OPO (set at 680 nm, Spectra-Physics, primoScan) was used as an excitation beam. The time-resolved decays were measured on a 9 stage PMT (Applied Photophysics) coupled with a monochromator set at 780 nm (Oriel Cornerstone 130, Newport), and the signals were collected by an oscilloscope (TDS 2022, Tektronix) connected to a computer. Nanosecond transient absorption (nsTA) measurements were performed using the same setup as used for time-resolved photoluminescence lifetime measurements with a quartz-halogen lamp as the probe light. The decays of nsTA were recorded by the PMT and the transient spectra were recorded on a CCD camera (iStar, Andor Technology). The STEM image was recorded on a Titan 80-300 TEM (FEI Co.) equipped with a monochromator, Cs probe-corrector, and Gatan image filter. The acceleration voltage of the TEM was 300 kV.

Femtosecond transient absorption

A Ti:sapphire oscillator (Tsunami, Spectra Physics) provides a seed beam centered at 800 nm for use in a regenerative amplifier (Spitfire, Spectra Physics) pumped by a frequency-doubled diode-pumped Nd:YLF laser (Evolution-X,Spectra Physics). The amplifier produces 800 nm pulses of around 200 fs duration (fwhm) at a 1 kHz repetition rate. The output from the amplifier was split and the two beams were used as probe and pump light. The 702 nm pump light was generated with an optical parametric amplifier (TOPAS, Light Conversion Ltd) and the power at the sample was 1 µJ/pulse. The probe light was generated with a CaF_2-plate and subsequently split into a probe beam and a reference beam, and the probe beam was overlapped with the pump at the sample. Pump-probe delay was adjusted by routing the pump beam from the OPA into a computer-controlled delay stage. The transmitted probe and reference beams were coupled to optical fibers and detected using a CCD camera (iXon-Andor).

HPLC

The photostationary state (PSS) of DAE after UV light irradiation (302 nm, 60 s) was determined with HPLC using a mobile phase of water and acetonitrile. Total run time was 20 min with an eluent ratio of 25% water in acetonitrile during 1-7 min, then 10% water in acetonitrile during 7-20 min.

Light irradiations

302 nm irradiation was performed using a UV analytic lamp (~10 mW cm^{-2} at the sample); and 523 nm irradiation (~15 mW cm^{-2} at the sample) was carried out using an LED light source (LED Engin, FWHM = 20 nm).

Cyclic voltammetry and spectroelectrochemistry

The cyclic voltammetry measurements were performed with a CHI-potentiostat controlled using CHI650A software (version 11.15). Platinum electrodes were used as the working and counter electrodes and an Ag/AgCl in saturated KCl was used as the reference electrode. The measurements were performed in thoroughly degassed

(using Argon) acetonitrile (HPLC grade, Fischer Scientific) with 0.1 M Tetra-n-butylammonium perchlorate TBAPF6 (Sigma Aldrich). Ferrocene/Ferrocenium (Fc/Fc^+) was used as an external standard with $E_{1/2}$ at approximately 0.435 V vs Ag/AgCl in CH_3CN.

The spectroelectrochemical measurements were performed under the same conditions as the cyclic voltammetry measurements with the exception that a honeycomb electrode with a Pt working- and counter electrode was used. An Avantes AVALIGHT-DHC was used as the light source and was directed at the honeycomb electrode using fiberoptic cables. The spectra were recorded with an Avantes (AvaSpec-2048) fiberoptic spectrometer.

Computational details

Density functional calculations (DFT) were performed using the Gaussian 16 software package.[3] Full optimization of the ground-state structure was performed by the hybrid functional B3LYP and basis set 6-31G(d). Excited state calculations were performed using the time-dependent formalism (TDDFT) and the basis set 6-31G(d,p).

2. Photochromic DAE

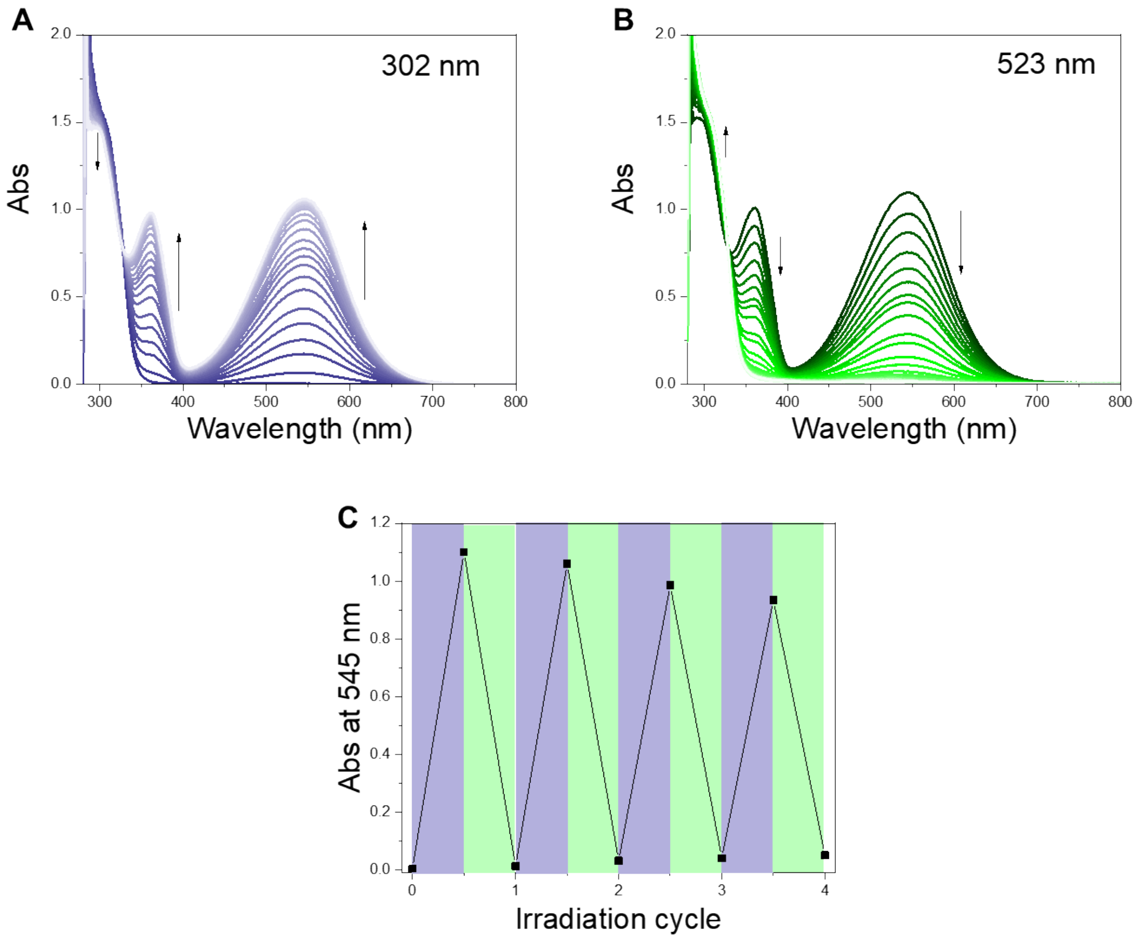

Figure S1. Light response of DAE in toluene. (A). UV-visible absorption spectra of 100 µM DAE-open under UV light (302 nm, 60 s) irradiation. (B). UV-visible absorption spectra of subsequent irradiation under green light (523 nm, 60 s). (C). Reversibility of the photoswitching of DAE evaluated by monitoring the absorbance at 545 nm over four irradiation cycles of 302 nm (60 s) and 523 nm light (60 s).

3. Steady-state characterization of DAE bound to PbS NCs

To isolate DAE covalently attached to PbS NCs, the general procedure of ligand exchange and washing step were carried out.[4-5] 1.5 ml of the mixed DAE (100 μM) and PbS NCs (1.5 μM) solution was precipitated in 8 ml of acetone, which can only dissolve DAE molecules but not PbS NCs. The solution was centrifuged at the speed of 8000 rpm for 5 min. The supernatant was discarded and the pellets of DAE anchored PbS NCs were re-dispersed in toluene. DAE bounded PbS NCs were purified using three cycles of repeated dispersion/precipitation/centrifugation, and finally dissolved in toluene under Argon atmosphere. As DAE molecules not anchored to PbS NCs are left in the supernatant, the above process can remove unbound DAE molecule and leave only the DAE bound to PbS NCs in the solution.

The number of DAE molecules bound to each PbS NC is determined by comparing the absorbances of each species,[6] i.e., DAE-closed molecules and PbS NCs in solution after three cycles of dispersion/precipitation/centrifugation, see Figure S2. Based on the red spectrum in Figure S2, and the molar extinction coefficient of DAE-closed 19 000 $M^{-1}cm^{-1}$at 545 nm and PbS NCs 135 000 $M^{-1}cm^{-1}$ at 750 nm, the number of anchored DAE-open molecules per PbS NC can be estimated to 35±5 DAE, taking into account of the composition at the PSS of DAE (90 % DAE-closed after UV light irradiation) and the measuring errors.

The above steady-state characterization implies that, when directly mixing with PbS NCs without further washing step, there is a fraction of DAEs that are not bound to the NCs, but instead free in solution. A dynamic equilibrium between this fraction and the bound fraction implies that we cannot exclude that at least part of the isomerization is ascribed to the non-bound fraction.

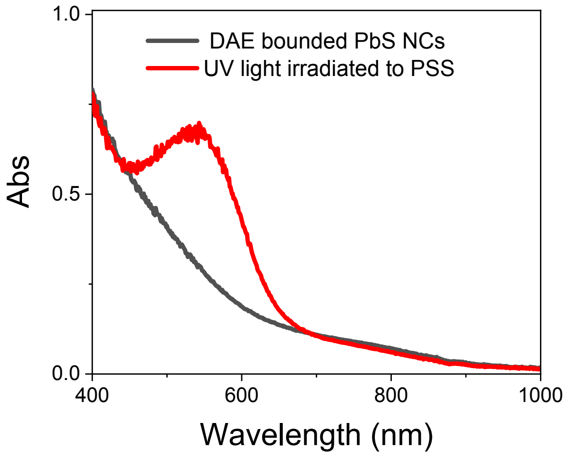

Figure S2. Absorption spectra of DAE bound to PbS NCs after three cycles of dispersion/precipitation/centrifugation (black), and after irradiation to the PSS using UV light (red).

4. Determination of the proportion of UV photon absorbed by DAEs in the mixture

The proportion of UV photons absorbed by DAE-open molecules in the mixture can be estimated by comparing the absorbances for DAE and PbS NCs. The absorbance of DAE-open in the mixture is 1.62 at 302 nm, however, PbS NCs have extremely strong absorption in the UV region, and it is therefore not possible to directly determine its absorbance at 302 nm. The UV-visible absorption spectrum of a 10-fold diluted PbS NCs alone solution (0.15

μM) was measured, whose absorbance is 1.01 at 302 nm. Therefore, the absorbance of 1.5 μM PbS NCs can be estimated to be 10.1 and the proportion of UV photons absorbed by DAE can be estimated to be ~14 %.

The kinetics of photoisomerization of DAE-open alone and PbS NCs mixed with DAE-open were also determined, see Figure S3. Monoexponential kinetics were used to fit the traces in Figure S3, which yielding a ring-closing rate $k_{ring\_closing} = 0.071\ s^{-1}$ for DAE-open alone and $k_{ring\_closing} = 0.011\ s^{-1}$ for DAE-open mixed with PbS NCs, respectively. This implies that the isomerization rate for DAE-open mixed with PbS NCs is 15.5 % of the corresponding rate without the PbS NCs, which is in good agreement with the above mentioned 14 % estimated from absorbance measurements. However, it cannot be excluded that isomerization is ascribed also to the fraction of DAE-open that is not bound to the NCs, but exist in a dynamic equilibrium with the bound fraction.

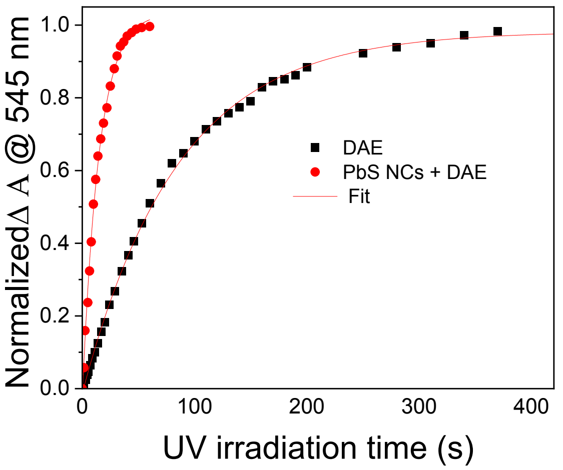

Figure S3. Evolution of the 545 nm absorbance of DAE-open (red circles) and PbS NCs mixed with DAE-open (black squares) under 302 nm irradiation over time. Red lines are monoexponential fits to the experimental data.

5. Photoluminescence lifetime measurement

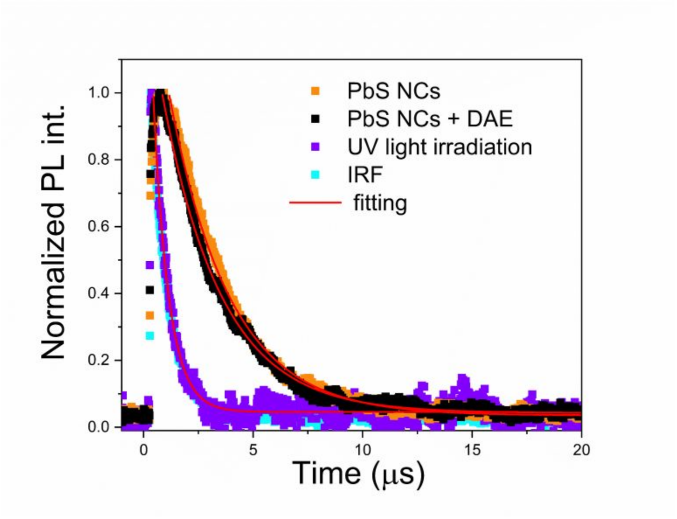

Figure S4. Time-resolved PL decays of PbS NCs (orange), PbS NCs and DAE-open (black), and after 302 nm irradiation (90 s, purple). The samples are excited at 680 nm, and the PL decays are recorded at 780 nm. Exponential decay fits are in red solid lines and the instrument response function (IRF, measured by a scattering sample) is in cyan.

6. Transient absorption decay traces

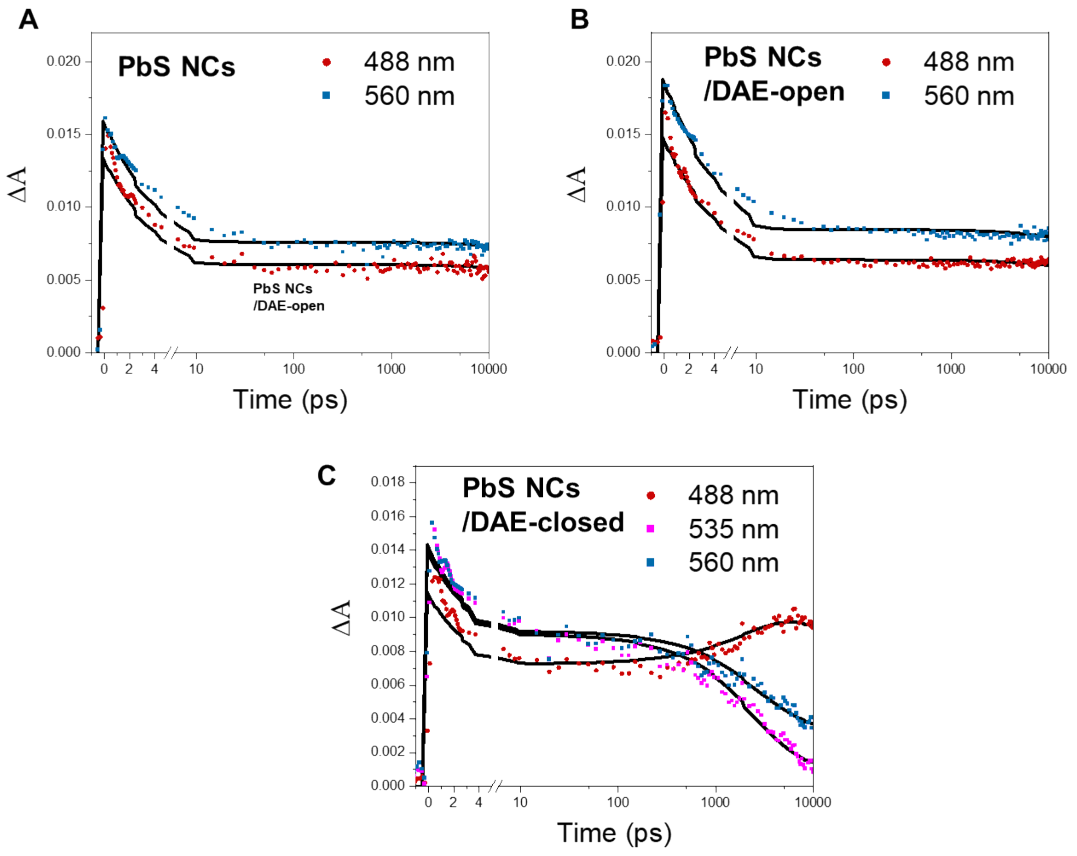

Figure S5. Transient absorption decay traces at a few selected wavelengths for (A) PbS NCs alone, (B) PbS NCs/DAE-open and (C) PbS NCs/DAE-closed. The measurements were performed in toluene with a pump pulse at 702 nm to selectively excite the PbS NCs.

7. Cyclic voltammetry of DAEs

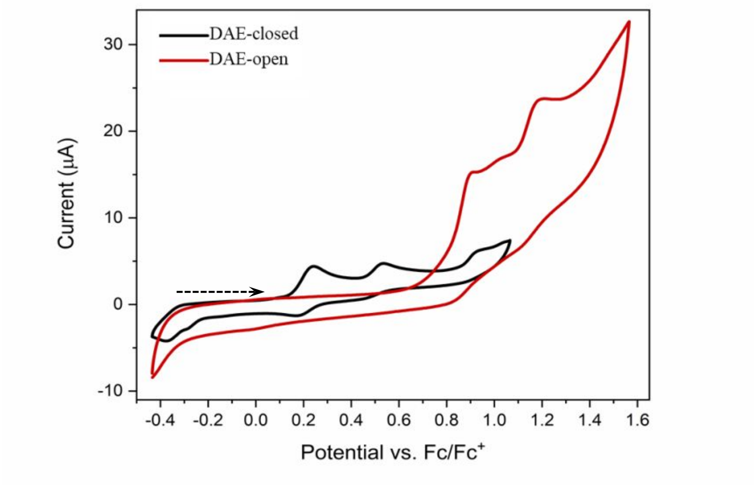

Figure S6. Oxidative CV curves of DAE-open and DAE-closed (0.5 mmol) in acetonitrile/0.1 M $TBAPF_6$ at room temperature at a positive scan rate of 0.1 V/s using a three electrode configuration.

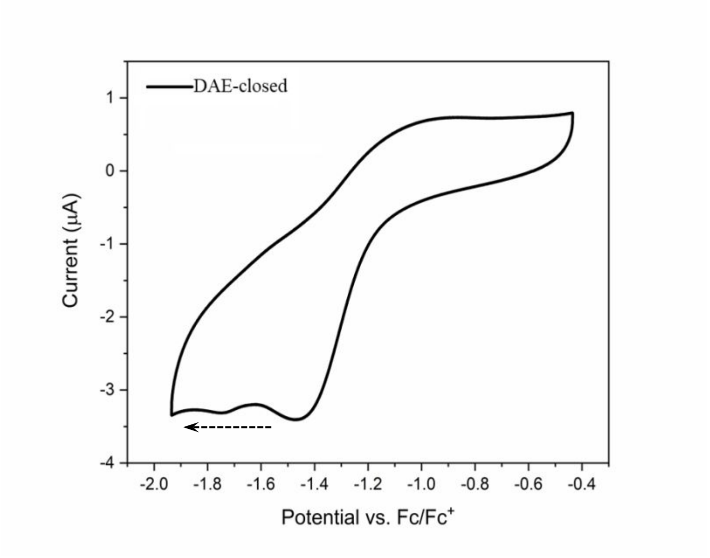

Figure S7. Reductive CV curve of DAE-closed (0.5 mmol) in acetonitrile/0.1 M $TBAPF_6$ at room temperature at a scan rate of 0.1 V/s using a three electrode configuration. The reductive scan of the open form is not presented here since the first reduction peak is outside the solvent window of acetonitrile.

8. Discussion on photoinduced electron transfer

The possibility of photoinduced electron transfer (PET) as the quenching mechanism was also investigated by means of cyclic voltammetry (Figure S6 and S7) and spectroelectrochemistry (Figure S9). From the Marcus-Rehm-Weller equation in combination with the Born dielectric continuum model of ion solvation, Eq. (1), the free energy change of a charge-separated ion pair can be estimated.[7-8]

$$\Delta G(D^{\bullet +} - A^{\bullet -}) = E_{ox}(D) - E_{red}(A) - \frac{e^2}{4\pi\varepsilon_0\varepsilon_s R_{DA}} + \left(\frac{e^2}{2}\right)\left(\frac{1}{r_D} + \frac{1}{r_A}\right)\left(\frac{1}{4\pi\varepsilon_0\varepsilon_s} - \frac{1}{4\pi\varepsilon_0\varepsilon_s^p}\right) - \Delta E_{00} \qquad (1)$$

Here, $E_{ox}(D)$ and $E_{red}(A)$ are the oxidation and reduction potentials of the donor and acceptor, respectively. e is the elementary charge, $\varepsilon_0(= 8.854 \cdot 10^{-12}\, As/Vm)$ is the permittivity of vacuum, ε_s is the static dielectric constant of the solvent the electrochemical measurements were performed in (acetonitrile) and ε_s^p is the dielectric constant of the solvent the photophysical measurements were measured in (toluene). The center-to-center donor acceptor distance R_{DA} was estimated as the sum of the radii r of PbS (15 Å) and the radii of the optimized structure of DAE (6 Å). ΔE_{00} is the electronic energy of the photoexcited PbS NCs, here estimated from the lowest energy absorption peak, 1.65 eV.

The two PET pathways that can quench the photoexcited state of PbS NCs is either PET from the LUMO of PbS NCs to the LUMO of DAE-closed or alternatively PET from the HOMO of DAE-closed to that of PbS NCs. The free energy change for both cases is presented together with the redox potentials in Table S1. The calculations clearly indicate that neither of the charge separated states ($PbS^{\bullet +}$ - DAE-closed$^{\bullet -}$ or $PbS^{\bullet -}$ - DAE-closed$^{\bullet +}$) are energetically accessible from the excited state of PbS in toluene for either the open or the closed form.

It should be noted that Eq. (1) is based on the interaction of two molecules in solution whereas the system investigated in this work is between NCs and molecules. Consequently, it is possible that Eq. (1) overestimates the solvent stabilization effect on the driving force due to the larger size of the PbS particles compared to a typical molecule. Indeed, studies on electron transfer rates to TiO_2 NCs of 50 nm in diameter have been shown to exhibit minimal dependence on the dielectric constant of the solvent.[9] However, as the particles become smaller and start to approach the size regime of larger molecules, as in the case of PbS under investigation here, there appears to be a substantial dependence on the dielectric constant of the solvent.[10] Wise et al. have shown that for PbS in the size regime of 3-4 nm there is a strong correleation between the dielectric constant of the solvent and electron

transfer rate.[11] They could on the other hand not find a strong correlation with solvent reorganization effect. Thus, it is clear that there exists a solvent dependence for the electron transfer, but that Eq. (1). might overestimate it slightly. However, since the driving force shown in Table S1 is substantially positive we conclude that even a smaller solvent effect should render the process non-spontaneous. For completeness, the HOMO and LUMO levels of PbS, DAE-open and DAE-closed vs. vacuum is shown in Figure S8. These values are based on CV measurements in acetonitrile and without accounting for the difference in solvent stabilization the electron transfer is only borderline spontaneous from the LUMO of PbS to the LUMO of DAE-closed.

Table S1. Oxidation and reduction potentials[a] of PbS NCs and DAE-open and DAE-closed as determined by cyclic voltammetry and calculated free energy changes, ΔG.[b]

	E_{ox} (V)	E_{red} (V)	ΔG (eV)
PbS	-0.03[c]	-1.68[c]	-
DAE-open	0.95	-3.15[d]	-
DAE-closed	0.25	-1.57	-
$PbS^{\bullet+}$ - DAE-open$^{\bullet-}$	-	-	2.11
$PbS^{\bullet-}$ - DAE-open$^{\bullet+}$	-	-	1.62
$PbS^{\bullet+}$ - DAE-closed$^{\bullet-}$	-	-	0.53
$PbS^{\bullet-}$ - DAE-closed$^{\bullet+}$	-	-	0.92

[a] All values are reported vs Fc/Fc^+. [b] Calculated using Eq. (1). [c] Values taken from literature.[12] [d] Estimated by adding ΔE_{00} to the oxidation potential since the reduction peak is outside the solvent window of acetonitrile.

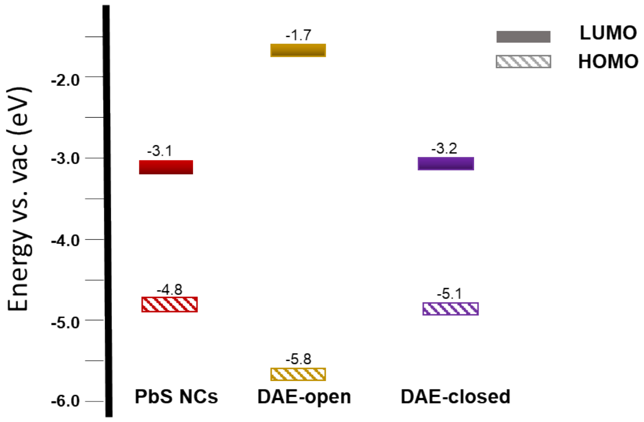

Figure S8. HOMO and LUMO levels of PbS NCs, DAE-open and DAE-closed. HOMO and LUMO energy levels of PbS NCs, DAE-open and DAE-closed as determined by cyclic voltammetry. Oxidation potentials in Table S1 are used to calculate the HOMO levels according to the equation $E^{HOMO} = -eE^{ox}(Fc/Fc^+) - 4.8\ eV$ where the offset of 4.8 eV corresponds to the ionization potential of ferrocene versus vacuum.[13] LUMO levels are estimated by adding the optical bandgap to the HOMO level.

9. Discussion on triplet spectroscopic signature of DAE-closed

Based on DFT calculations, TET is energetically favorable from PbS NCs to DAE-closed, but not to DAE-open. To conclusively establish that TET is the main quenching mechanism, nsTA measurements of PbS NCs mixed with DAE-closed have been performed using 410 nm pulsed excitation (2 mJ/pulse), however, no long-lived decay was observed at the excited state absorption (ESA, ~560 nm) on the timescale of microsecond. We also made attempts to independently determine the spectroscopic signature of the T_1-T_n transition of DAE-closed using triplet sensitization and subsequently compare it to the spectra obtained using fsTA in Figure 3. Zinc octaethyl

porphyrin (ZnOEP, E(T_1) ~1.78 eV) and Platinum octaethylporphyrin (PtOEP, E(T_1) ~1.9 eV) were used in an attempt to directly generate the triplet spectrum of DAE-closed. The sensitizers were excited at 405 or 536 nm with a pulse width of 10 ns and 1.5 mJ/pulse. Despite the substantial driving force for triplet energy transfer owing to the high triplet energy of the sensitizers we were not able to obtain the triplet spectra of DAE-closed. We confirmed, using phosphorescence quenching measurements of PtOEP in the presence and absence of DAE-closed that DAE-closed does indeed efficiently quench the excited state of the sensitizers. It is possible that the triplet state of the closed form is very short lived and thus cannot reach concentrations high enough to be observed with the instrumentation available during sensitization measurements. To the best of our knowledge, there is no reported triplet spectra of the closed form of DAE derivatives in the literature. Since the results from the triplet sensitization attempts were inconclusive, TDDFT calculations were performed to support the hypothesis regarding the TET mechanism. The calculated T_1-T_n transitions and their corresponding oscillator strengths for DAE-closed optimized in the ground state are presented in Table S2. It is known that TDDFT calculations are sensitive to the choice of functional and basis set, and the experimental data of excitation energy here does not match exactly with the calculated values. However, the purpose of the performed TDDFT calculation was to get a qualitative assignment of the observed triplet spectrum, and the results show that there are indeed allowed transitions in 450-650 nm region shown in Figure 3.

Since the triplet excited state absorption spectra of the closed form could not be obtained through sensitization, spectroelectrochemistry was employed to compare the spectroscopic signature of the reduced and oxidized species of DAE-closed with spectral component C from the fsTA measurements in Figure 3, which corresponds to the acceptor state that quenches the PL from the PbS NCs. The results from the spectroelectrochemical measurements are presented in Figure S9 together with component C from Figure 3.

Figure S9 clearly shows that the radical cation of the closed form ($DAE - closed^{\bullet +}$, blue trace) does not match component C from the fsTA measurements in Figure 3. This indicates that PET from DAE-closed to PbS NCs after photoexcitation is likely not the main reason for the observed PL quenching. The radical anion ($DAE - closed^{\bullet -}$, green trace) partly overlaps with component C in the 480 nm region, and thus PET from PbS NCs to DAE-closed may occur. However, based on the lack of driving force discussed in section 6 in addition to the mismatch in the 575 nm region, we conclude PET is not the main mechanism of current system and the spectroscopic features of component C is mainly from the triplet state of DAE-closed.

Table S2. Calculated T_1-T_n transition energies and oscillator strengths for DAE-CLOSED

	**Excitaiton energy (eV)**	**Wavelength (nm)**	**Oscillator strength**
T_1-T_2	1.11	1117	0.0033
T_1-T_3	1.97	631	0.3587
T_1-T_4	2.34	530	0.0135
T_1-T_5	2.48	499	0.0705
T_1-T_6	2.51	493	0.0384
T_1-T_7	2.72	455	0.0983

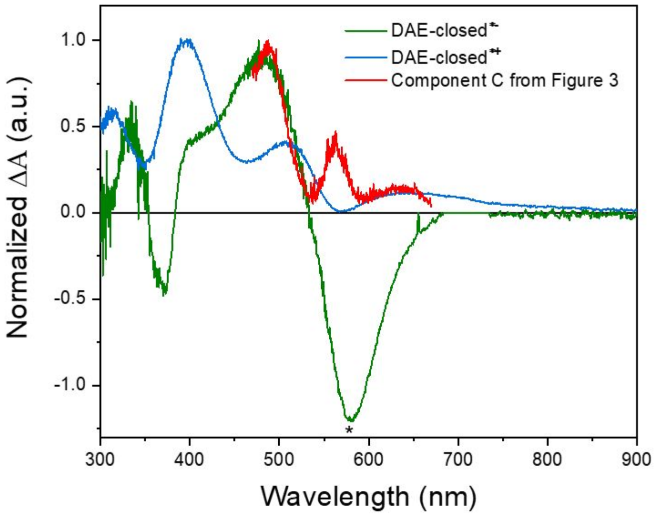

Figure S9. Differential absorption spectra of the radical anion (green) at obtained at -1.9 V vs Fc and the radical cation (blue) obtained at +1.1 V vs Fc. The differential aborbance is obtained by substraction of the ground state spectra, i.e. without applying any potential. Component C from the fsTA measurements in Figure 3 is also presented (red), which corresponds to the excited state of DAE-closed that forms due to either TET or ET from PbS. *The negative band of the radical anion centered at roughly 580 nm corresponds to the loss of ground state absorption of the closed form when forming the radical anion and possibly also from switching induced by the probe light.

10. References

(1) Hines, M. A.; Scholes, G. D., Colloidal PbS nanocrystals with size-tunable near-infrared emission: Observation of post-synthesis self-narrowing of the particle size distribution. *Adv. Mater.* **2003,** *15* (21), 1844-1849.

(2) Liu, T. Y.; Li, M. J.; Ouyang, J. Y.; Zaman, M. B.; Wang, R. B.; Wu, X. H.; Yeh, C. S.; Lin, Q.; Yang, B.; Yu, K., Non-Injection and Low-Temperature Approach to Colloidal Photoluminescent PbS Nanocrystals with Narrow Bandwidth. *J. Phys. Chem. C* **2009,** *113* (6), 2301-2308.

(3) Frisch, M. J., Gaussian 16, Revision C.01e. **2016**.

(4) Garakyaraghi, S.; Mongin, C.; Granger, D. B.; Anthony, J. E.; Castellano, F. N., Delayed Molecular Triplet Generation from Energized Lead Sulfide Quantum Dots. *J. Phys. Chem. Lett.* **2017,** *8* (7), 1458-1463.

(5) Bender, J. A.; Raulerson, E. K.; Li, X.; Goldzak, T.; Xia, P.; Van Voorhis, T.; Tang, M. L.; Roberts, S. T., Surface States Mediate Triplet Energy Transfer in Nanocrystal-Acene Composite Systems. *J. Am. Chem. Soc.* **2018,** *140* (24), 7543-7553.

(6) Mongin, C.; Garakyaraghi, S.; Razgoniaeva, N.; Zamkov, M.; Castellano, F. N., Direct observation of triplet energy transfer from semiconductor nanocrystals. *Science* **2016,** *351* (6271), 369-372.

(7) Kilså, K.; Macpherson, A. N.; Gillbro, T.; Mårtensson, J.; Albinsson, B., Control of electron transfer in supramolecular systems. *Spectrochimica Acta Part A: Molecular and Biomolecular Spectroscopy* **2001,** *57* (11), 2213-2227.

(8) Weller, A., Photoinduced Electron Transfer in Solution: Exciplex and Radical Ion Pair Formation Free Enthalpies and their Solvent Dependence. *Zeitschrift für Physikalische Chemie* **1982,** *133* (1), 93-98.

(9) Hyun, B.-R.; Bartnik, A. C.; Sun, L.; Hanrath, T.; Wise, F. W., Control of Electron Transfer from Lead-Salt Nanocrystals to TiO2. *Nano Lett.* **2011,** *11* (5), 2126-2132.

(10) Zhu, H.; Yang, Y.; Wu, K.; Lian, T., Charge Transfer Dynamics from Photoexcited Semiconductor Quantum Dots. *Annu. Rev. Phys. Chem.* **2016,** *67* (1), 259-281.

(11) Hyun, B.-R.; Bartnik, A. C.; Lee, J.-K.; Imoto, H.; Sun, L.; Choi, J. J.; Chujo, Y.; Hanrath, T.; Ober, C. K.; Wise, F. W., Role of Solvent Dielectric Properties on Charge Transfer from PbS Nanocrystals to Molecules. *Nano Lett.* **2010,** *10* (1), 318-323.

(12) Hyun, B.-R.; Zhong, Y.-W.; Bartnik, A. C.; Sun, L.; Abruña, H. D.; Wise, F. W.; Goodreau, J. D.; Matthews, J. R.; Leslie, T. M.; Borrelli, N. F., Electron Injection from Colloidal PbS Quantum Dots into Titanium Dioxide Nanoparticles. *ACS Nano* **2008,** *2* (11), 2206-2212.

(13) Pommerehne, J.; Vestweber, H.; Guss, W.; Mahrt, R. F.; Bässler, H.; Porsch, M.; Daub, J., Efficient two layer leds on a polymer blend basis. *Adv. Mater.* **1995,** *7* (6), 551-554.
